# Using Carbon Paste Electrode Modified with Ion Imprinted Polymer and MWCNT for Electrochemical Quantification of Methylmercury in Natural Water Samples

**DOI:** 10.3390/bios12060376

**Published:** 2022-05-30

**Authors:** Ruddy Mesa, Sabir Khan, Maria D. P. T. Sotomayor, Gino Picasso

**Affiliations:** 1 Laboratory of Physical Chemistry Research, Faculty of Sciences, National University of Engineering, Av. Tupac Amaru 210, Lima 15333, Peru; ruddy.mesa.l@uni.pe (R.M.); gpicasso@uni.edu.pe (G.P.); 2 Institute of Chemistry, São Paulo State University (UNESP), São Paulo P.O. Box 14801-970, Brazil; skhan@uni.edu.pe

**Keywords:** methylmercury quantification, IIPs, electrochemical sensor

## Abstract

Methylmercury (MeHg) is one of the most toxic organic mercury compounds found in the environment. The continuous exposure of human beings to this highly toxic compound may damage their nervous system. The present work reports the development and application of a novel electrochemical sensing technique for the quantification of MeHg using a modified carbon paste electrode with multi-walled carbon nanotubes (MWCNTs) combined with ion imprinted polymer, which is highly selective toward MeHg (CPE/MWCNTs/IIP-MeHg) detection. The ion imprinted polymer was synthesized using 2-mercaptobenzothiazole (MBT), acrylic acid (AA) and MeHg employed as ligand, functional monomer and template ion, respectively, and the synthesized material was characterized by Raman spectroscopy and SEM-EDX. Both the proposed and control sensors were characterized by cyclic voltammetry (CV) and electrochemical impedance spectroscopy (EIS). The electrochemical measurements were carried out using differential pulse stripping voltammetry (DPSV), and a well-defined anodic peak observed at about +0.138 V (vs. Ag/AgCl) was recorded for MeHg. The application of the CPE/MWCNTs/IIP-MeHg sensor (which increased the charge transfer on the electrode surface) under the DPSV-based electrochemical method (which enhanced the signal intensity) made the detection technique highly sensitive and selective for the quantification of methylmercury. Under optimum experimental conditions, the proposed sensor exhibited a linear response range of 560–610 µg L^−1^ and a detection limit of 0.538 µg L^−1^, with acceptable relative error values ≤1% when applied for the detection of MeHg in real water samples.

## 1. Introduction

The Methylmercury cation (MeHg) is an organic mercury form, which has been found to be much more toxic compared to inorganic Hg [1,2]. Methylation of inorganic Hg is an important process that takes place in aquatic environments; this process occurs in both fresh water and seawater under oxygen-limited conditions and its occurrence largely depends on the activity of anaerobic microorganisms [3]. Once MeHg is released from microbes, the cation can be concentrated through the food chain as a consequence of its high solubility in lipids [4]. The continuous exposure of human beings to MeHg can seriously impair the nervous system, particularly in pregnant women where it can lead to irreversible damages to the central nervous system of the unborn child [5]. In general, methylmercury can be more toxic than inorganic mercury, and despite its high toxicity level, there has not yet been an established safe threshold level of MeHg in drinking water due to the scarcity of data on this highly toxic compound and the absence of laboratories that are capable of performing an efficient test for its quantification in water. The consumption of contaminated water and seafood has been found to be the primary source of non-occupational exposure to MeHg. Owing to the high toxicity of organic mercury, the quantification of total mercury in water is found to be insufficient for determining the toxicological impacts of methylmercury on biota and human health. Thus, the development of a simple method for the determination of methylmercury is essentially important and has become a hot topic of research in recent times.

Voltammetry is a widely popular analytical technique; the attractiveness of this technique lies in the portability of its instrumentation, low costs, and good sensitivity [6]. Heaton and Laitinen initially investigated the electroanalytical reduction in MeHg in aqueous solution using pulse polarography and cyclic voltammetry [7]. After that, other research was conducted on electrochemical sensors for MeHg using the following materials: manganese dioxide (MnO_2_)/gold nanoparticles (AuNPs) composites/glassy carbon electrode [8]; AuNPs/zeolitic imidazolate framework/glassy carbon electrode [9]; AuNPs/reduced graphene oxide modified/glassy carbon electrode [6]; AuNPs/graphitic carbon nitride/glassy carbon electrode [10], etc. More recently, studies reported in the literature have employed ion imprinted polymers (IIPs)-based electrochemical sensors targeted at improving the selectivity of the sensing device in the determination of heavy metals due to specific binding sites to the easy access for analytes [11,12]. The combined use of polymers and carbon nanomaterials in the construction of electrochemical sensors leads to the development of sensing devices with excellent sensitivity, selectivity, and stability [13].

The present work reports the development and application of a highly selective and sensitive sensor for the quantification of MeHg. The sensor was developed using a carbon paste electrode modified with an imprinted polymer, which is highly selective toward MeHg (this was reported in our previous study [14]) and multi-walled carbon nanotubes-MWCNTs (which increase the charge transfer on the electrode surface). The electrochemical determination of MeHg was conducted by differential pulse stripping voltammetry (DPSV). To the best of our knowledge, this study is the first report of its kind in the literature where IIPs were employed for the quantification of MeHg ions through the application of a voltammetric sensor.

## 2. Materials and Methods

All the chemicals employed for the preparation of the stock and standard solutions used in the experiments were of analytical grade. A standard solution of 1000 µg L^−1^ of methylmercury chloride (CH_3_HgCl) was purchased from Alfa Aesar (São Paulo, Brazil). Electrolyte solutions were prepared using ultrapure water (with resistivity of 18.2 MΩ cm) along with hydrochloric acid (HCl) and Ethanol 96%—both acquired from Merck. Tin stannous chloride (SnCl_2_. 2H_2_O), potassium ferricyanide/ferrocyanide (Fe(CN_6_)^3−/4−^), potassium chloride (KCl), graphite powder (GP), multiwall carbon nanotube (MWCNTs), and mineral oil were obtained from Sigma-Aldrich (Maryland, USA). Standard solutions of mercury (II), cadmium (II), lead (II) and hierro (II) were used for the conduct of selectivity analyses—all these solutions were acquired from MERCK (São Paulo, Brazil).

The ion imprinted polymer for methylmercury (IIP-MeHg) and its corresponding non-ion imprinted polymer-NIP (absence of the analyte) were synthesized via bulk polymerization using MeHgCl as the template, 2-mercaptobenzothiazole (MBT) as the ligand, acrylic acid (AA) as the functional monomer, ethylene glycol dimethacrylate (EGDMA) as the cross-linker and ethanol as the porogenic solvent. More details about synthesis and characterizations are in our previous paper [15].

All the voltammograms were obtained based on the application of a typical three-electrode system, which consisted of the following: Ag/AgCl (KCl 3 mol L^−1^) used as a reference electrode; platinum wire used as an auxiliary electrode; and a carbon paste electrode (CPE) modified with ion imprinted polymer, which is selective toward methylmercury (IIP-MeHg), used as the working electrode. Voltammetric measurements were conducted using a DropSens µSTAT 400 Bipotentiostat (Oviedo, Spain) with the aid of a Dropview 8400 software. The total quantification of Hg was performed according to the EPA Method 245.1 [15] using PerkinElmer FIMS 400 mercury analyzer (Maryland, USA); this was conducted in order to verify the accuracy of the electrochemical results.

The electrochemical quantification of MeHg was performed by differential pulse stripping voltammetry (DPSV) using 0.05 mol L^−1^ HCl (pH 1.0) solution. For the pre-concentration procedure, the deposition potential applied on the working electrode was −0.8 V (vs. Ag/AgCl) for 500 s under stirring (500 rpm). After 10 s of equilibration, the voltammogram was recorded under the following conditions: scanning potential range: −0.15 to 0.4 V; amplitude: 0.05 V; pulse width: 0.05 s; potential step: 4 mV; and pulse period: 0.5 s. Prior to the conduct of the subsequent DPSV test, the electrode surface was cleaned through the application of a potential of −0.8 V for 300 s under stirring in a chloride-free solution containing a strong complexing agent, 0.1 mol L^−1^ KSCN; this was done in order to remove any traces of mercury [11].

Several water samples were collected from different places in Peru: Cañete river (Lima, Peru), Humay water treatment plant (Pisco, Peru), and tap water (Lima, Peru). The water samples were collected in cleaned plastic bottles with a capacity of one liter and acidified to pH 1 at the sampling site. All samples were analyzed within 48 h of collection. Prior to analysis, the samples were filtered with Whatman 42 paper to remove any solid materials. The final samples were stored in the fridge at 5 °C.

## 3. Results

### 3.1. Morphological and Chemical Characterization of IIP-MeHg and NIP

**Figure 1:**
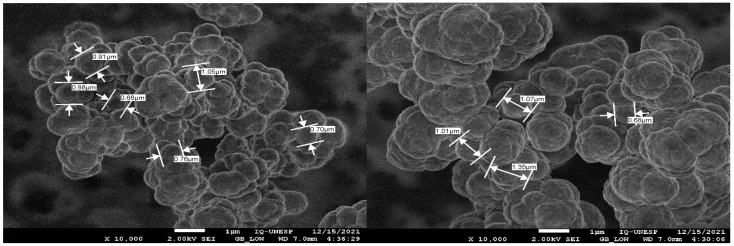
SEM micrographs of (**left side**) IIP-MeHg and (**right side**) NIP (magnification: 10,000×).

The surface morphology (SEM images) of the ion imprinted polymer for methylmercury (IIP-MeHg) and the non-ion imprinted polymer (NIP) is shown in Figure 1. Both the IIP-MeHg and NIP particles produced were found to be uniform in shape and size; this may be attributed to the bulk polymerization method, which yields sorbents with good adsorption capacity and small particles—this was confirmed by the Brunauer-Emmett-Teller (BET) surface analysis of IIP-MeHg and NIP conducted previously [16]. Another reason that could explain the particles uniformity in shape and size may be associated with the use of ethanol as solvent for the synthesis of polymers since it provides good porosity. The mean size of the IIP-MeHg particles (<1 μm) was found to be slightly lower than that of the NIP (>1 μm); this shows that the template ion exerts a significant influence over the growth of imprinted particles during the process of polymerization since the MeHg template acts as a nucleon and attracts the monomers toward it, thus decreasing the size of the IIP particles.

The amount of the main chemical constituent present in the polymer backbone was estimated by energy-dispersive X-ray spectroscopy (EDS) and Raman spectroscopy. A significant amount of oxygen and carbon observed in both polymers (see Figure S1) may be attributed to the presence of a great quantity of the cross-linker EGDMA. On the other hand, a small amount of sulfur, observed in the IIP-MeHg, may be linked to the selective ligand present in the ion imprinted polymer. Furthermore, as can be observed in Figure S2, the strong absorption band at 2959 cm^−1^ is related to CH_2_ groups and indicates the completion of the polymerization process in both the imprinted and non-imprinted samples.

### 3.2. CV and EIS Characterization

**Figure 2:**
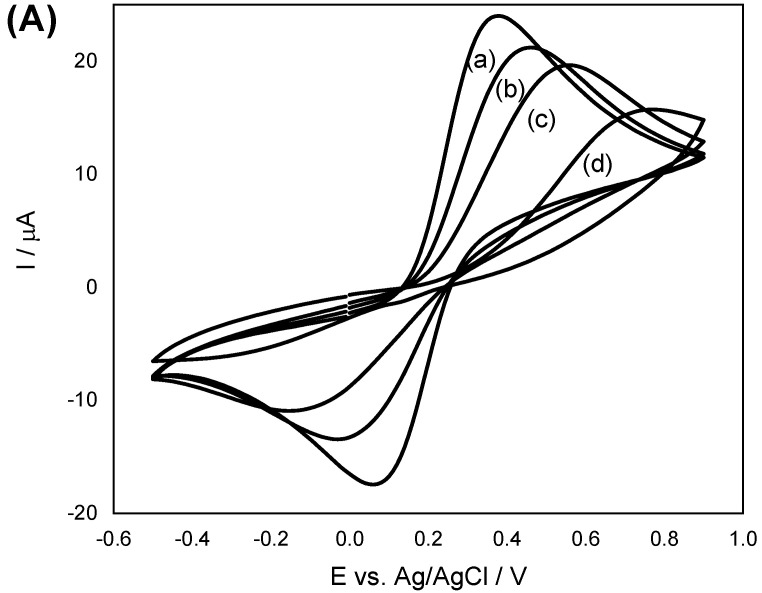

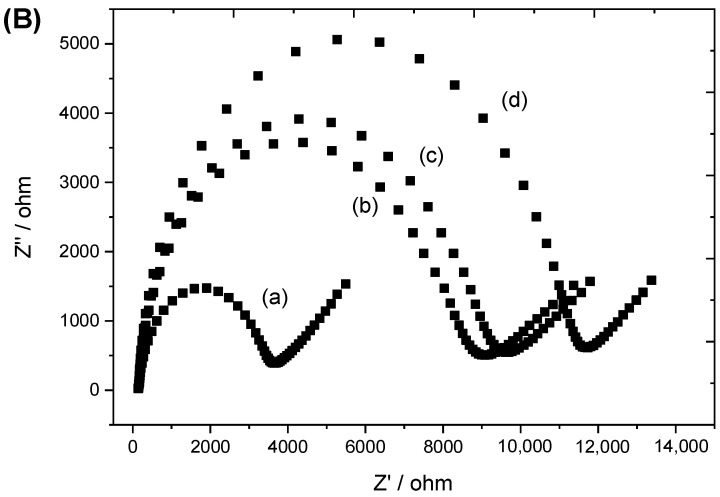
(**A**) CV and (**B**) Nyquist plots related to EIS characterization of (a) CPE, (b) CPE/MWCNTs/IIP-MeHg, (c) CPE/MWCNTs/NIP and (d) CPE/IIP-MeHg applied in 2.5 mmol L^−1^ [Fe(CN) _6_]^3–^, 2.5 mmol L^−1^ [Fe(CN)_6_]^4–^ and 0.1 mol L^−1^ KCl.

Cyclic voltammetry (CV) and electrochemical impedance spectroscopy (EIS) curves were obtained from the application of four different working electrodes in 2.5 mmol L^−1^ Fe(CN_6_)^3−^, 2.5 mmol L^−1^ Fe(CN_6_)^4−^, and 0.1 mol L^−1^ KCl solutions (Table S1). As shown in Figure 2A, CV curves obtained for all the electrodes were nearly symmetrical and were characterized by reversible redox reactions. The redox peaks of CPE/IIP-MeHg were considerably lower than those of the bare CPE as the polymer material in the electrode surface caused a disruption in the electron transfer rate. A comparative analysis of the electrodes showed that the CPE/MWCNTs/IIP-MeHg and CPE/MWCNTs/NIP exhibited an increase in peak currents, owing to the multi-walled carbon nanotubes (MWCNTs) present in them, which was used as a modifier; the presence of the MWCNTs enhanced the electrode performance by increasing the velocity of the electron transfer. The EIS technique was used to study the interface property of the modified electrodes. Looking at the EIS curves, the semicircle portion at high frequencies is related to the electron transfer resistance while the linear portion at low frequencies reflects diffusion [17]. As can be observed in Figure 2B, the CPE/IIP-MeHg modified electrode exhibited an increase in electron transfer resistance compared to the bare CPE; this outcome is attributed to the isolated properties of the polymers, which is corroborated by the CV curves. The MWCNTs modified electrodes-CPE/MWCNTs/IIP-MeHg and CPE/MWCNTs/NIP, exhibited a decrease in electron transfer resistance due to their good electrical conductivity. The combined properties of IIP-MeHg and MWCNTs led to the production of an electrode with low electron transfer impedance.

### 3.3. Voltammetric Behaviour of MeHg on CPE

The cyclic voltammetry of methylmercury chloride on carbon paste electrode (CPE) was investigated in acidic medium. The data obtained from this analysis were found to be very similar to those obtained by Heaton and Laitinen [7] and Afonso et al. [18]. The mechanism involving the electrochemical reduction in MeHg on the working electrode is suggested to be as follows:

(1) $${\mathrm{CH}}_{3}{\mathrm{Hg}}^{+}+e^{-}\;↔{\;\mathrm{CH}}_{3}{\mathrm{Hg}}^{.}$$

(2) $$2{\;\mathrm{CH}}_{3}{\mathrm{Hg}}^{.}↔\;{\left({\mathrm{CH}}_{3}\mathrm{Hg}\right)}_{2}↔\;{\left({\mathrm{CH}}_{3}\right)}_{2}\mathrm{Hg}+\mathrm{Hg}$$

(3) $${\mathrm{CH}}_{3}{\mathrm{Hg}}^{.}+{\;H}^{+}+1e^{-}\;↔{\;\mathrm{CH}}_{4}+\;\mathrm{Hg}$$

Figure S3 illustrates the reduction in MeHg. The first cathodic peak, peak C1 at −0.48 V, represents the reduction in MeHg to methylmercury radical CH_3_Hg·, then, the second cathodic peak, peak C3 at −0.69 V, represents the reduction in CH_3_Hg· to Hg. It is worth noting that CH_3_Hg· can be disproportionate to (CH_3_Hg)_2_ within a short time [14]; these reactions occur on the electrode surface during deposition. During the anodic scan, the stripping peak current correspondent to peak C1 is not observed; instead, one notices the presence of an anodic peak A2 at more positive potential, around 0.21 V, which indicates the oxidation of Hg to Hg^2+^. The data clearly show that all the methylmercury has been converted to Hg^2+^. Furthermore, the intensity of the anodic peak A2 appears to be unchanged with the number of scanning cycles; this is thus a well-defined, reproducible peak for measuring methylmercury.

### 3.4. Optimization of Experimental Parameters

**Figure 3:**
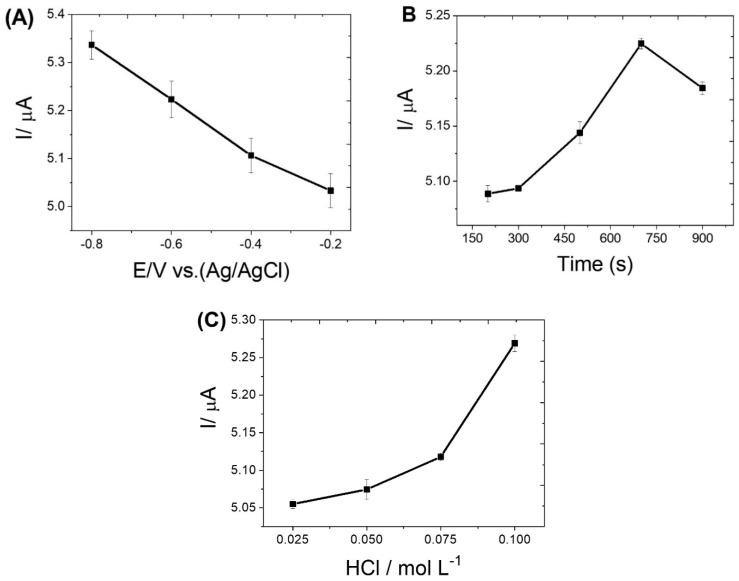
The influences of: (**A**) pre−concentration potential; (**B**) pre−concentration time; and (**C**) hydrochloric acid concentration on the peak current of 600 µg L^−1^ MeHg.

The electrochemical responses of MeHg based on the application of the modified carbon paste electrodes were evaluated by differential pulse stripping voltammetry (DPSV). Figure S4 shows that the CPE/MWCNTs/IIP-MeHg sensor exhibited higher response for MeHg compared to the bare CPE and CPE/MWCNTs/NIP sensors, which did not have selective cavities for methylmercury. The results obtained show that the synergies between the electronic transport property of MWCNTs and the high selectivity of IIP-MeHg toward MeHg mean that more MeHg could be reduced and deposited on the electrode surface during the pre-concentration process. The proposed CPE/MWCNTs/IIP-MeHg sensor was used for a rapid electrochemical quantification of methylmercury. To obtain an optimal performance of the CPE/MWCNTs/IIP-MeHg sensor, a thorough analysis was conducted with a view to investigating the dependence of the DPSV peak current on the preconcentration potential, preconcentration time, and hydrochloric acid concentration—such as the supporting electrolyte. As shown in Figure 3A, the biggest peak current of MeHg was obtained at a potential of −0.8 V. Thus, −0.8 V was chosen as the optimal potential for the reduction in MeHg; this potential was applied in further studies. Figure 3B shows that the MeHg redissolution current increases linearly with the preconcentration time (200–700 s). After a period of 700 s, the MeHg redissolution current decreases due to the possible saturation of the electrode surface; in view of that, 500 s was selected as the preconcentration time. The HCl concentration was evaluated in the range of 0.025–0.1 mol L^−1^, where a linear dependence of the redissolution current was observed (Figure 3C). At above the concentration of 0.5 mol L^−1^, the current experiences a sharp decrease, possibly as a result of the formation of calomel (Hg_2_Cl_2_) or other insoluble mercury compounds on the surface of the electrode. Thus, 0.05 mol L^−1^ HCl was chosen as the optimal concentration.

### 3.5. Analytical Performance

**Figure 4:**
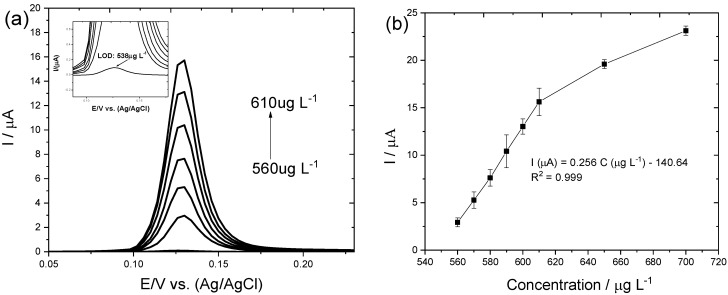
(**a**) DPSV responses related to the increase in MeHg concentration and inset the limit detection figure; (**b**) Calibration plot for methylmercury determination at 50 V/s. The amounts of MeHg concentration determined were 560, 570, 580, 590, 600, and 610 µg L^−1^. Each point was an averaged value of three peak currents obtained from background-subtracted voltammograms.

Figure 4a shows the DPSV responses related to the increase in MeHg concentration and Figure 4b shows the calibration plot in the concentration range of 560–610 µg L^−1^. The relationship between the average peak current I in µA and concentration C in µg L^−1^ is given by the regression equation: Ip (µA) = 0.256 C (µg L^−1^)–140.64 (n = 3, R^2^ = 0.999). As the MeHg concentration increases, the response of the CPE/MWCNTs/IIP-MeHg sensor is found to decrease; this outcome can be attributed to the increase in the dimerization rate and the tendency of competition that occurs with the oxidation of methylmercury radical. The limit of detection based on the lowest concentration of an MeHg that can be consistently detected as different to the signal-to-noise ratio (S/N = 3) was calculated to be 538 µg L^−1^. Table 1 presents a comparative analysis of the CPE/MWCNTs/IIP-MeHg sensor proposed in this study and other electrochemical sensors reported in the literature used for methylmercury determination. As can be noted, compared to the other sensors previously reported in the literature, the CPE/MWCNTs/IIP-MeHg sensor exhibited a short linear range due to the fact that at lower concentrations, the electrochemical signal of MeHg is not detectable and at higher concentrations MeHg is absorbed on the surface of the electrode, impairing subsequent measurements.

The precision of the proposed sensing method in terms of repeatability was evaluated using seven consecutive electrochemical measurements with MeHg concentration of 590 µg L^−1^, in addition, for the reproducibility, the experiments were performed in two different potentiostats (DropSens µSTAT 400 and µSTAT-i 400s). The relative standard deviation obtained from these analyses were 0.98% and 1.98%, which in both cases were lower than RDS_Horwitz_—see Table S2. The reusability and stability of the CPE/MWCNTs/IIP-MeHg sensor was investigated by DPSV using a solution of 590 µg L^−1^ MeHg for seven days. The results obtained from this analysis are shown in Figure S5, and after six days of experiments, the current was found to decrease from 10.60 µA to 10.43 µA. These results point to a good stability of the proposed sensor and its suitability for reutilization

### 3.6. Interference Studies

**Figure 5:**
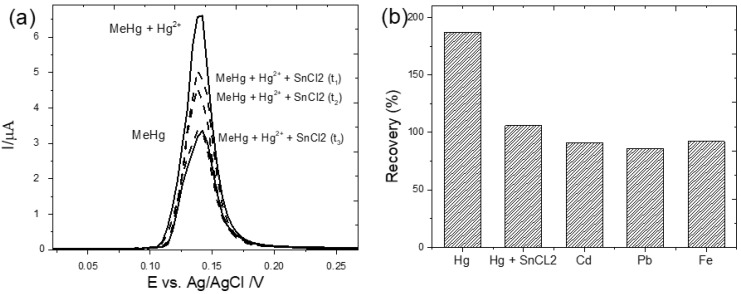
(**a**) DPSV responses obtained for MeHg in the presence of Hg^2+^ and SnCl_2_ at different times of pretreatment (dash line) and without pretreatment (solid line); (**b**) the effect of other metal ions on the peak current of 600 µg L^−1^ MeHg based on the application of the CPE/MWCNTs/IIP-MeHg sensor.

To obtain more accurate determinations of MeHg, one needs to investigate and eliminate any possible interference from other compounds when it comes to the application of the proposed sensing device for the determination of MeHg in matrices of interest. The inorganic Hg^2+^ molecule coexists with MeHg in natural water; thus, the former can cause a non-negligible interference when it comes to MeHg determination as a result of the overlap of the stripping currents. Previous studies have shown that inorganic Hg^2+^ and MeHg reacted differently with SnCl_2_ (which has been found to only reduce inorganic Hg^2+^) [11,12,13]. For the interference analysis, a study was conducted in order to investigate the reduction in Hg^2+^ by SnCl_2_. Figure 5a shows the pretreatment analysis performed through the application of 2.4 mmol L^−1^ SnCl_2_.2H_2_O under vigorous stirring for 5 (t1), 10 (t2) and 20 min (t3). As can be seen, the influence of Hg^2+^ was removed in 20 min with a 99% recovery rate.

A thorough analysis was also carried out in order to study the chemical interference derived from the traces of other toxic metal ions in MeHg quantification using Cd^2+^, Pb^2+^, Fe^3+^ and Hg^2+^ in the presence and absence of SnCl_2_. To conduct this interference analysis, the concentrations of MeHg and the possible interferents were employed in the ratio of 1:1 at 600 µg L^−1^. As can be seen in Figure 5b, the sample containing SnCl_2_ exhibited a recovery rate of 106%, while the sample without SnCl_2_ presented a recovery rate of 187%. With regard to the other interferents, the effect of Cd^2+^, Pb^2+^, and Fe^3+^ on MeHg detection was negligible since the recovery percentage was around 100%. Based on these results, it can be concluded that the metal ions do not exert any competing influence when it comes to the application of the proposed sensor for methylmercury quantification.

### 3.7. Application in Natural Water Samples

An analysis was carried out in order to determine the applicability of the CPE/MWCNTs/IIP-MeHg sensor for the quantification of MeHg in three water samples: tap water, Cañete river water, and water from the water treatment plant in the Humay district of Pisco in Peru. The results obtained from this analysis showed that the samples did not contain MeHg (CVAAS, LD 0.5 µg L^−1^). Table 2 shows the relative errors (%) with respect to the CVAAS reference method applied for total mercury analysis. In all the cases evaluated, the relative error values obtained were ≤1%; thus, there is no significant difference between the proposed method and the reference method in terms of MeHg quantification. Essentially, the proposed method employed for the quantification of MeHg was found to be as efficient as the CVAAS technique; this finding points to the efficiency and convenience of the voltammetric approach when applied to the conduct of electrochemical analyses.

## 4. Discussion

The present study reported the development and application of the novel electrochemical sensor CPE/MWCNTs/IIP-MeHg for the quantification of methylmercury in water samples by differential pulse stripping voltammetry (DPSV) under optimized conditions. The results pointed to the important role played by imprinting and selective specific cavities in the polymer structure when it comes to the application of the proposed sensor for the detection of MeHg ions. The interference of inorganic Hg^2+^ in MeHg detection was minimized by the use of SnCl_2_, and the proposed sensor exhibited good selectivity toward MeHg and satisfactory repeatability for the quantification of MeHg. Table 1 shows the most recent electrochemical sensors reported in the literature for the determination of MeHg. As can be seen, CPE/MWCNT/IIP-MeHg presented LD = 538 µg L^−1^ and higher linear ranges than the other sensors, since concentrations below 560 µg L^−1^ did not show any measurement current. However, the proposed method could be used in future studies for the analysis of methylmercury in food samples such as fish, since it usually presents high concentration of MeHg (0.5 mg Kg^−1^ for fish products and fish meat and 1.0 mg Kg^−1^ for mercury-accumulating fish).

## 5. Conclusions

The CPE/MWCNTs/IIP-MeHg sensor proposed in this study is cheap and simple to construct in the lab, in addition, it also has a good repeatability and stability of the proposed sensor using the same experimental condition. The novel sensor was constructed for the first time using some simple available and low-cost material and does not require any particular skill or sophisticated instruments. This new analytical method shows a better identification, good selectivity, high affinity, low cost, and easy preparation and has good stability. The sensing device has proved to have excellent application potential for the quantitative determination of MeHg in natural water samples.

## Figures and Tables

**Table 1:** Summary of previous electrochemical sensors used for methylmercury determination.

Material	Voltammetric Technique	Linear Range (µg L^−1^)	Material
GCE/AuNPs-RGO	DPSV	646–5175	[6]
GCE/AuNPs/mpg-C_3_N_4_	DPSV	215–5390	[10]
Glassy Carbon Microelectrodes	LSV	64–646	[17]
CPE/MWCNT/IIP-MBT-AA	DPSV	560–610	The present work

**Table 2:** Determination of methylmercury in water samples using the CPE/MWCNTs/IIP-MeHg sensor. For comparison purposes, MeHg concentration was also determined using the reference method: CVAAS (S/N = 3).

Sample	Added MeHg (µg L^−1^)	Method		Relative Error % ^1^
		MeHg Detected Using CVAAS (µg L^−1^)	MeHg Detected Using DPSV with CPE/MWCNTs/IIP-MeHg (µg L^−1^)	
Tap water	590	615.2 ± 0.9	609.8 ± 1.18	−0.88
River water	590	609.5 ± 1.46	598.5 ± 1.62	−1.80
Eluent from a treatment plant	590	596.5 ± 1.1	594.5 ± 2.42	−0.34

^1^ Relative error % = (proposed method − standard method/standard method) × 100.

## Data Availability

Not applicable.

## Supplementary Materials

The following supporting information can be downloaded at https://www.mdpi.com/article/10.3390/bios12060376/s1: Figure S1. EDS images of (a) IIP-CH_3_Hg^+^ and (b) NIP; Figure S2. Raman spectrum of (a) IIP-CH_3_Hg^+^ and (b) NIP; Figure S3. Cyclic voltammetry of CPE applied in a solution containing 1000 µg L^−1^ CH_3_Hg^+^ and 0.05 mol L^−1^ HCl, recorded at 0.1 V s^−1^; Figure S4. DPSV responses obtained from the application of 1000 µg L^−1^ CH_3_Hg^+^ in 0.05 mol L^−1^ HCl solution for (a) CPE/MWCNTs/IIP-CH_3_Hg^+^, (b) CPE/MWCNTs/NIP and (c) CPE electrodes. Deposition potential: −0.8 V (vs. Ag/AgCl); deposition time: 500 s; Figure S5. (a) Analysis of repeatability and (b) reusability of the proposed sensor using CH_3_Hg^+^; Table S1. Composition of working electrodes prepared in this work; Table S2. Estimation of the experimental RSD and Horwitz to evaluate the precision in terms of repeatability and reproducibility.
