# Supplementary material for: Using Carbon Paste Electrode Modified with Ion Imprinted Polymer and MWCNT for Electrochemical Quantification of Methylmercury in Natural Water Samples
Source: Biosensors (Basel). 2022 May 30;12(6):376. doi: 10.3390/bios12060376 (PMC9221305; doi:10.3390/bios12060376)
Supplement: Supplementary file 1 [file biosensors-12-00376-s001.zip › biosensors-1728760-supplementary.pdf]

Supplementary Materials

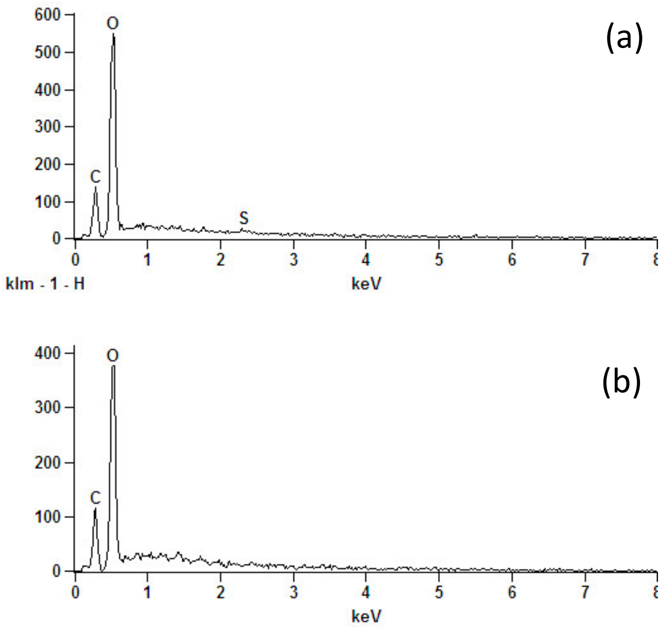

Figure S1. EDS images of (a) IIP-CH_3Hg^+ and (b) NIP.

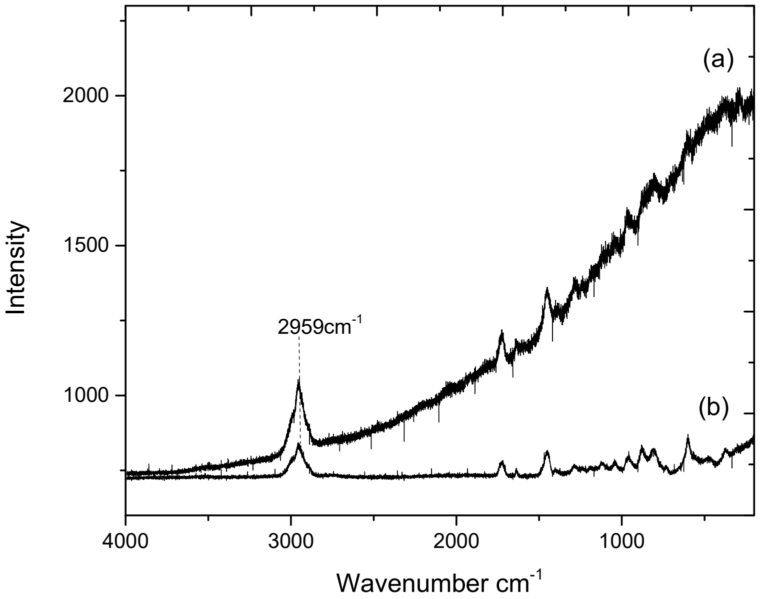

Figure S2. Raman spectrum of (a) IIP- CH_3Hg^+ and (b) NIP.

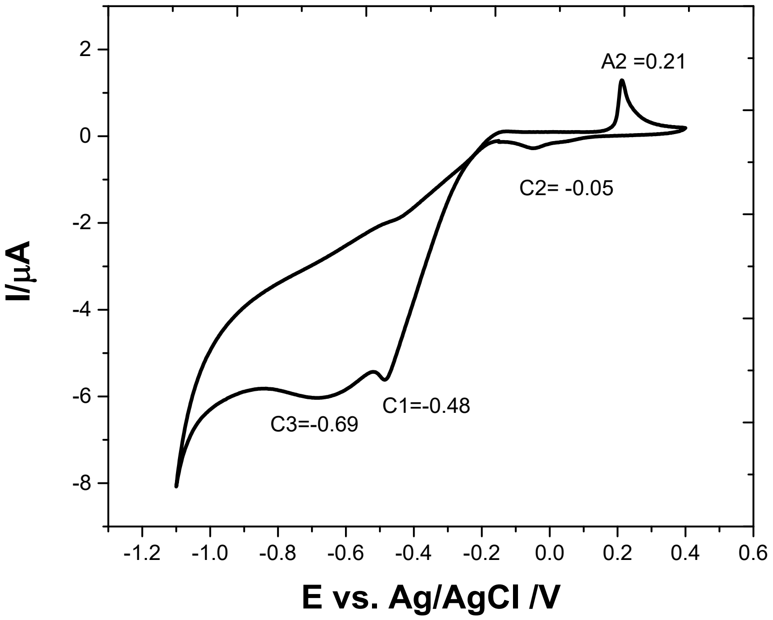

Figure S3. Cyclic voltammetry of CPE applied in a solution containing 1000 µg L^{-1} CH_3Hg^+ and 0.05 mol L^{-1} HCl, recorded at 0.1 V s^{-1}

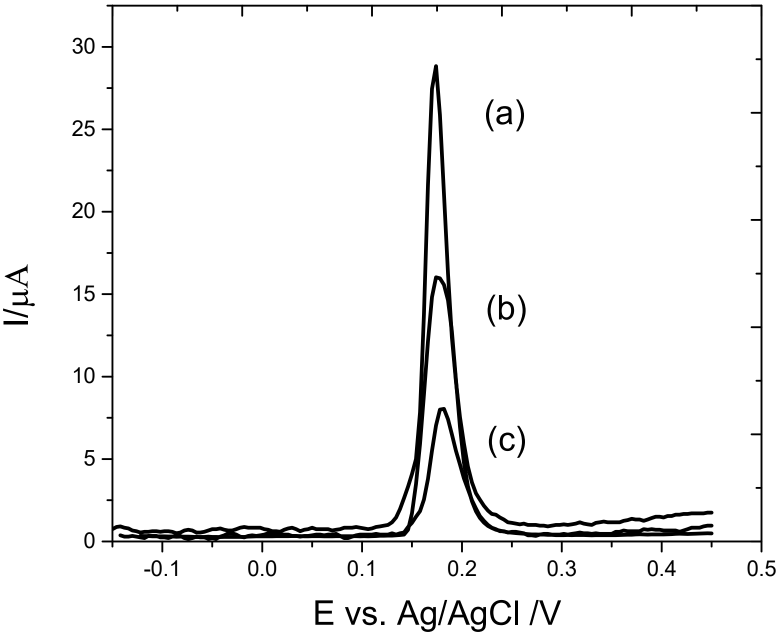

Figure S4. DPSV responses obtained from the application of 1000 μg L^{-1} CH_3Hg^+ in 0.05 mol L^{-1} HCl solution for (a) CPE/MWCNTs/IIP- CH_3Hg^+, (b) CPE/MWCNTs/NIP and (c) CPE electrodes. Deposition potential: -0.8 V (vs. Ag/AgCl); deposition time: 500 s.

Table S1. Composition of working electrodes prepared in this work.

Nomenclature	Composition
**CPE**	Graphite (100%)
**CPE/IIP- CH_3Hg^+**	Graphite (80%), IIP- CH_3Hg^+ (20%)
**CPE/MWCNTs/IIP- CH_3Hg^+**	Graphite (70%), MWCNTs (10%), IIP- CH_3Hg^+ (20%)
**CPE/MWCNTs/NIP**	Graphite (70%), MWCNTs (10%), NIP (20%)

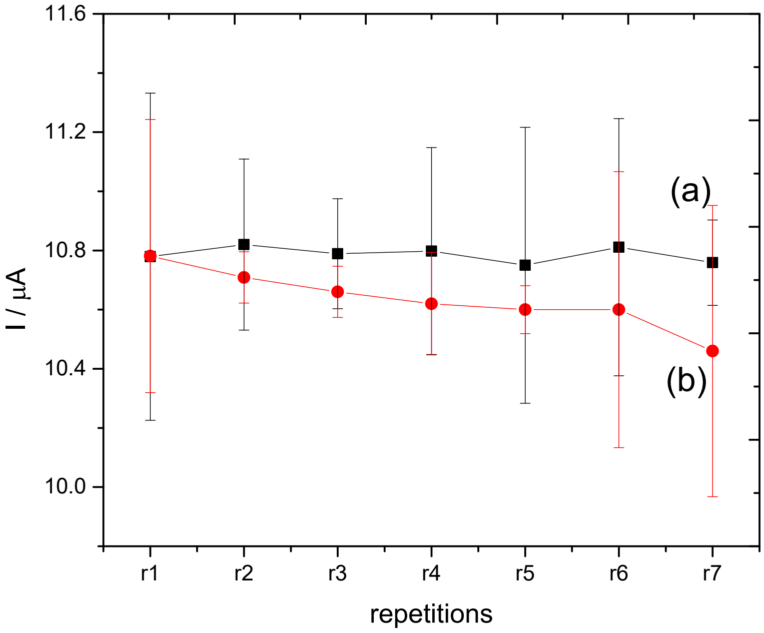

Figure S5. (a) Analysis of repeatability and (b) reusability of the proposed sensor using CH_3Hg^+

Table S2. Estimation of the experimental RSD and Horwitz to evaluate the precision in terms of repeatability and reproducibility

	**repeatability**	**reproducibility**
**$\%RDS_{experimental}$[1]**	**0.981**	**1.981**
**$\%RDS_{Horwitz}$[2]**	**9.14**	**3.179**

$\%RSD_{experimental}$[1]= (standard deviation) *100/ Mean

$\%RSD_{Horwitz}$[2]= $2\ (2^{1-0.5(\text{Log concentration})})/3$
